# Agent-Object Relationships in Level-2 Visual Perspective Taking: An Eye-Tracking Study

**DOI:** 10.5334/joc.398

**Published:** 2024-10-10

**Authors:** Ben Ford, Rebecca Monk, Damien Litchfield, Adam Qureshi

**Affiliations:** 1 University of Gloucestershire, UK; 2 Edge Hill University, UK

**Keywords:** visual perspective taking, level-2, agent object relationships, object congruency, intentionality, eye-tracking, stereotype

## Abstract

Visual perspective taking (VPT) generates a shared frame of reference for understanding how the world appears to others. Whilst greater cognitive and neurophysiological demands are associated with increasing angular distance between the self and other is well documented, accompanying attentional characteristics are not currently understood. Furthermore, although age and group status have been shown to impact task performance, other important cues, such as the relationship between agents and objects, have not been manipulated. Therefore, 35 university students participated in an eye-tracking experiment where they completed a VPT task with agents positioned at a low or high angular disparity (45° or 135° respectively). The congruence between the age of the agent (child vs adult) and the object they are attending to (e.g., teddy-bear vs kettle) was also manipulated. Participants were required to respond to the direction of the object from the agent’s position. The findings reveal more fixations and increased dwell-times on agents compared to objects, but this was moderated by the age of the task agent. Results also showed more attentional transitions between agents and objects at higher angular disparities. These results converge with behavioural and neurophysiological descriptions of task performance in previous studies. Furthermore, the congruency of the relationship between agents and objects also impacted attention shifting and response times, highlighting the importance of understanding how social cues and contexts can modulate VPT processes in everyday contexts and social interaction.

## Introduction

Representing the visual perspectives of others is a fundamental sociocognitive process. Evidence supports a distinction between the ‘level 1’ computations which are fast, involuntary, and required for visibility judgments (L1-VPT; Samson et al., 2010), and those for ‘level 2’, which are slower and involve a deliberate mental translation into the other’s position in order to represent *how* the world appears visually or spatially (L2-VPT; Flavell et al., 1981; Kessler & Thomson, 2010; Kessler & Rutherford, 2010; Kessler & Wang, 2012). Behavioural and neurophysiological studies of L2-VPT demonstrate that the computations required depend on the task agent’s degree of rotation away from a shared line-of-sight (Kessler & Thompson, 2010). At lower angles, participants can use their egocentric reference frame because the self and other perspectives are broadly similar. However, with angular disparity above 80°, participants perform a mental self-rotation into the position of the other person (Kessler & Thompson, 2010; Surtees et al., 2013a; Surtees et al., 2013b). This rotation required at higher angular disparities increases cognitive effort and is also evident in greater neurophysiological activity across a broad frontoparietal network (Seymour et al., 2018). However, despite cognitive and neurophysiological evidence, whether there are differences in attention allocation between low and high angular disparities is yet to be understood.

To-date, no eye-tracking experiments have been conducted with L2-VPT, yet eye-tracking data from L1-VPT and related processes such as gaze-cueing reveal significant effects of task manipulations. For example, work on gaze-cueing reveals preferential attention to in-group others (for a review see Kawakami et al., 2018) whether determined by minimal groups (Kawakami et al., 2014, study 2), race (Wu et al., 2012; Kawakami et al., 2014, studies 1 and 3), or age (Denkinger & Kinn, 2018; Strickland-Hughes et al., 2020). As for L1-VPT, when participants need to switch between self and other perspectives, compared to when perspective cues remained constant (e.g., always ‘YOU’ trials), participants seem to experience interference from the task agent’s differing perspective (Ferguson et al., 2017). Ferguson et al. (2017) also reveal a greater number of fixations on switching trials and suggested that the broader visual search required may modulate this type of interference. Further, Cane et al. (2017) found that under high working memory load and when perspectives differed, participants made earlier fixation shifts towards a cued object, shifts which were not evident when perspectives were shared. This suggests that participants were incorporating their privileged knowledge about the perspective of the other into subsequent processing. Whilst eye-tracking research reveals significant effects of agent cues (Kawakami et al., 2018), perspective cues (Ferguson et al., 2017) and cognitive load (Cane et al., 2017), no research has examined attentional allocation in a L2-VPT task, nor how this may be moderated by angular differences to the task agent’s position. Thus, the first aim of this study is to provide converging evidence for the distinction between low and high angular judgements by assessing whether there are differences in the number of fixations, the number of transitions between the object and agents, and the proportion of time spent looking at either the object or agent. Doing so will supplement our current understanding of cognitive and neurophysiological mechanisms in L2-VPT.

Understanding the influence of social cues on perspective representation is also gaining increasing attention. To date, VPT has been shown to be affected by social factors such as group status (Savitsky et al., 2011; Todd et al., 2011; Simpson & Todd, 2017; Ferguson et al., 2018), humanness of agents (Ye et al., 2020) and avatar emotion (Monk et al., 2020). For example, divergent findings either report that similarity to task agents facilitates perspective representation (Ye et al., 2020) or creates egocentric interference (Savitsky et al., 2011; Simpson & Todd, 2017; Todd et al., 2011; Ferguson et al., 2018). More generally, these results suggest that the other person in VPT tasks is being considered as a social agent, with their attributes exerting a top-down effect on representing visual perspectives. However, while the extant literature to date has employed VPT tasks that present agents and their attentional-targets (e.g., the letters, objects or room features the agents are ‘looking’ towards), no research has examined whether the relationship between the agent and attentional-target impacts subsequent processing or attention allocation.

A close thematic relationship between agent and object (e.g., ‘doctor’ and ‘stethoscope’) results from the cooccurrence of one or multiple semantic features which are processed by a general representational mechanism (Xu et al., 2018). These thematic relationships are represented quickly and implicitly, are based on first-hand experience of objects and events (Estes et al., 2011; Chaigneau et al., 2009; Kalénine et al., 2012) and have been shown to support learning, memory, and language comprehension (Estes et al., 2011). Therefore, it is possible that a close thematic relationship could also facilitate VPT performance. Furthermore, the close relationship between an agent and object could be a salient indicator of intentionality (Estes et al., 2011). For example, Chaigneau et al. (2009) found that compared to when objects are presented alone (e.g., projectiles), participants’ ability to respond with the object’s function is improved by providing contextual cues (e.g., a catapult) along with information about how they will be used. Intentionality judgments are important here because they come under the umbrella of Theory-of-Mind (ToM; Leslie, 1987) which plays a fundamental role in successful communication (Weiss & Harris, 2001; Hughes & Leekam, 2004; Brüne, 2005) and has previously been related to VPT (Hamilton et al., 2009; Dumontheil et al., 2010). In fact, observing others not only reveals their intentions but allows us to predict their future interaction with the environment (Frith & Frith, 2006) and shared intentionality is considered by some as the basis of ToM (Tomasello, 2018).

Crucially for VPT tasks, where an agent typically looks at an object in front of them, Becchio et al’s (2008) review concluded that gaze alone can lead to the ascription of intentionality from an agent to an object. This ‘intentional imposition’ occurs when the target of another’s gaze is endowed with properties it does not inherently show and is inferred from social affordances which arise from a combination of the automatic activations evoked by perceiving objects (object affordances, e.g., a toy affords being played with) and a context or intentional action (e.g., reaching for the toy; Gong et al., 2021). Whilst dynamic actions embedded in contexts yield a greater haemodynamic response in premotor mirror neurons (Iacoboni et al., 2005), an actual movement or vocalisation is not necessary to mentalise about others’ intentions; gaze alone is sufficient (Castiello, 2003; Teufel et al., 2010a; Teufel et al., 2010b; Morgan et al., 2018; Morgan et al., 2021; although, see Cole et al., 2015 and Ward et al., 2020). Therefore, visual perspective tasks where participants simply view an agent looking at objects provide a befitting paradigm to manipulate inferred intentionality without having to make this potential action explicit (Nummenmaa & Calder, 2009). Furthermore, research examining semantically incongruent information, such as viewing a microscope on a kitchen worktop, has been related to attentional measures such as the number and duration of fixations (Henderson et al., 1999; Võ & Henderson, 2009). Yet, it remains unknown whether thematic relationships influence attention allocation in L2-VPT. As VPT requires attentional reorienting, incorporating eye-tracking to reveal the attentional impact agent-object relationships is pertinent and represents a novel and significant contribution to the existing literature on L2-VPT.

In summary, this pre-registered study (https://osf.io/8gdm6) project uses an established L2-VPT task manipulating angular disparity of the task agent whilst also manipulating whether the agent is an adult or child and whether the object attended is adult or child related. It seeks to understand whether the age of a task agent or agent-object relationship significantly affect cognitive demand, as measured by response times and accuracy, and gaze-behaviour, as measured by agent-object dwell-time proportion, overall number of fixations, and attentional transitions between objects and agents. Furthermore, eye-tracking will extend the current evidence that supports distinctive processing at lower and higher angular disparities to include attention allocation.

## Hypotheses

### Low vs High Angular Disparities

Replicating previous behavioural findings (Michelon & Zacks, 2006; Kessler & Thompson, 2010; Surtees et al., 2013a; Surtees et al., 2013b), we hypothesise that perspective representation at 135° compared to 45° will result in significantly slower response times and lower accuracy rates. To extend current understanding on attentional allocation in L2-VPT, we predict perspective judgements at 135° will require more fixations, more area-of-interest transitions (AOITs) between the agent and target object, and greater proportional focus on the avatar.

### Avatar-age

Extending previous ingroup effects on VPT (Savitsky et al., 2011; Todd et al., 2011; Simpson & Todd, 2017; Ferguson et al., 2018; Ye et al., 2020), we expect the visual perspective of adult (ingroup) avatars to be more easily represented, leading to significant quicker response times and significantly less fixations required to process the visuospatial information. We do not expect any avatar-age effects on accuracy, AOITs or dwell-times.

### Object congruence

We hypothesise that congruent age-object relationships will result in significantly quicker response times (akin to Xu et al., 2018), less fixations (akin to Henderson et al., 1999; Võ & Henderson, 2009), significantly less AOITs, and less time spent looking at the object relative to the agent. We do not hypothesise any accuracy effects.

### Interaction effects

To supplement the pre-registered hypotheses, and for clarity, there is currently no evidence for any outcome measure to hypothesise an interaction between angular disparity and object congruence, angular disparity and avatar-age, object-congruence and avatar-age, or a three-way interaction.

## Method

### Participants

35 participants (27 female) from XXXXX participated for course credit or were recruited via advertisements placed around campus and paid £5.00. 1 participant was removed due to low overall accuracy (<66%). The final sample included 34 participants (26 female; *M*age = 21.06, *SD*age = 4.33, Range = 18–35). As per the pre-registration, recruitment was restricted by budget and time but was in line with previous research using similar design and dependent measures (Fergusson et al., 2017). As per Westfall et al. (2014) with a stimuli-within-condition design, a power of .8 and 34 participants, the current experiment was powered to detect a main effect of *d* = .50.

### Design

**Figure 1:**
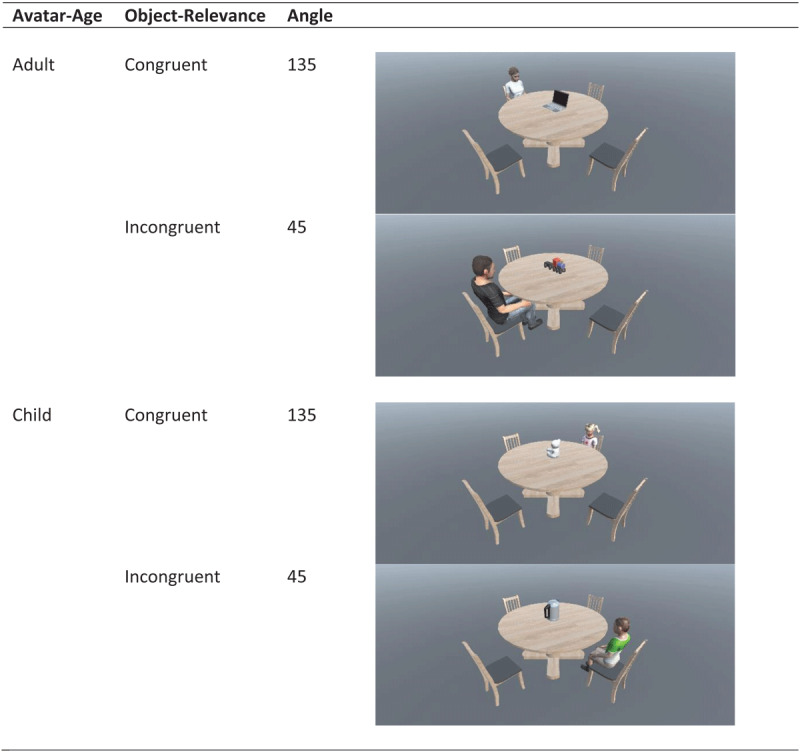
Example stimuli to demonstrate manipulations of avatar-age, object congruence and angular disparity.

A 2 × 2 × 2 repeated measures design with three independent variables of angle (low vs high), avatar-age (child vs adult) and object-relevance (relevant vs irrelevant) as factors. Example stimuli can be seen in Figure 1. Dependent measures included accuracy, response times, number of fixations, area of interest transitions and proportionate dwell time.

### Materials

Participants completed an adapted version of the Kessler and Thomson (2010) task. Stimuli were designed using Unity game engine (Unity Technologies, 2020) and the experiment was programmed in Experiment Builder (SR Research). Original stimuli dimensions were 1920 × 1080. Stimuli portrayed a circular table from above, with the sight-angle set at 35°. At the table were 4 chairs, positioned at 45°, 135°, 225°, and 315°. 0° was the line-of-sight of participants and angular disparity was determined by their rotation around an imaginary arc that borders the target object in the centre of the table. On each stimulus, a human avatar was seated at one of these positions. The avatars gender always matched the participant’s gender. Realistic human avatars were used because the distinction between children and adults was integral to the manipulation. Each stimulus had a single object placed in the middle of the table. This object could be child-related (a teddy-bear or toy train), or adult related (a laptop or kettle). These objects were selected because of their spatial properties. Participants were told that left-facing meant a teddy-bear’s face pointing leftward, a train’s locomotive pointing leftward, a laptop screen facing leftward, and a kettle’s spout on the left-side as this is the direction it would pour (see Figure X). Across all stimuli, the child and adult sat in each of the tables positions equally, they were also paired with each object an equal number of times, and all had an equal number of leftward and rightward facing correct responses. All stimuli are available at https://osf.io/8gdm6.

### Procedure

**Figure 2:**
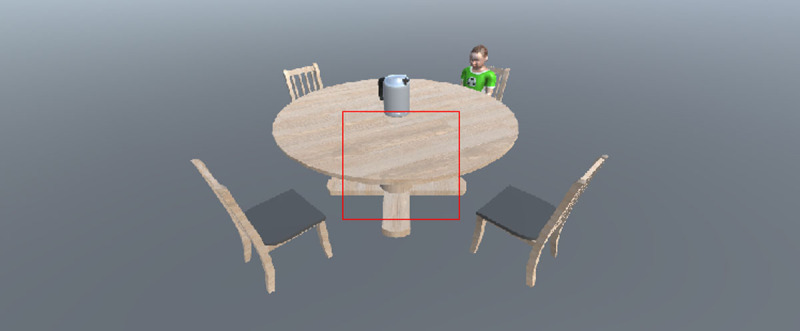
The red square represents 5° of visual angle from the centre of the screen. The target objects and avatar positions are located beyond the boundaries to encourage eye-movements.

Participants rested their heads on a fixed chin rest 60 cm from the 19 inch (360 × 270 mm) ViewSonic Graphic Series G90fB coloured display screen. The left eye of participants was recorded using an EyeLink 1000+ eye-tracker running at 1000 Hz. Participants were shown example images of the objects facing left and right (see Figure X for some examples). Participants were then shown example experimental stimuli (see Figure X) and told that they had to respond to whether the object was facing right or left from the avatars position using the ‘Q’ for leftward and ‘P’ for rightward. Each trial began with a fixation cross lasting 750 ms and was followed by the experimental stimuli that remained on screen until a response was made or 8 seconds had elapsed. The experimental stimuli were 1024 × 460 pixels and appeared centrally on the screen; this corresponds to 33.4 × 15.35° of visual angle which should mean participants had to move their gaze to focus on the individuals sat at the seating positions (a graphic with the standard 5° of visual angle (149.03 × 149.03 pixels) laid over an example stimulus can be seen in Figure 2). Participants completed 8 practice trials, before completing 128 experimental trials (16 trials per condition) presented randomly across two equal blocks. Participants completed a standard EyeLink 9-point calibration at the beginning of the experiment, after the practice trials and in the break between the two blocks of trials.

### Eye-tracking Measures

On each trial, a total of two *Area of Interest Transitions (AOIs)* were specified; one surrounding the object with a constant size of 82 × 82 pixels for all objects and one surrounding the task agents which at higher angular disparities was 86 × 123 pixels and at lower angles 144 × 185 pixels. AOITs are measured as the number of times participants fixated in one AOI before leaving the AOI boundary and fixating in the other AOI. For example, if participants fixated on the agent AOI, shifted attention outside the AOI boundary and fixated on something other than the object AOI before returning to the agent AOI, this would not count as a transition. However, if participants fixating on the agent AOI shifted attention outside the boundary and then fixated on the object AOI, this would count as one AOIT; if they immediately shifted back to the agent AOI, this would count as a second AOIT. *Proportionate dwell time* is calculated as time fixating on avatar AOI subtracted from total time fixating within object AOI, divided by total trial time and multiplying the outcome by 100. Thus, it acts as a proportionate measure of attention allocation between task agents and task objects relative to total trial time, with positive values indicating greater attention to objects and negative values indicating greater attention to agents. *Total number of fixations* are the sum of all fixations irrespective of where in the scene they were made. Thus, total fixations were not restricted to AOI fixations and therefore inherently linked to response times.

## Results

Factors of angle (45°/135°), avatar-age (child/adult) and age-object relevance (relevant/irrelevant) were entered into a linear mixed effects model for response times, number of fixations, area-of-interest transitions (AOITs) and proportionate dwell times, and logistic regression for accuracy data using the lme4 package (Bates et al., 2015) for R (R Core Team, 2013). Random intercepts were included to control for variation across individuals and stimuli,^1^ reducing the potential for type 1 error that can arise if they are treated as fixed (Barr, 2013; Barr et al., 2013). For mixed effects models on number of fixations and area of interest transitions, maximal models and models including a random effect of stimuli led to models with a singular fit. Therefore, for these models, only random effects of individual were included. Post-hoc tests were computed using the ‘emmeans’ package (Lenth et al., 2019) for R and multiple comparisons were controlled using a Bonferroni adjustment. All means and standard deviations for each outcome measure can be found in Table 1 and all statistical outcomes in Tables 2 and 3.

**Table 1:** Means and standard deviations on all dependent measures for all conditions.


INDEPENDENT VARIABLES			DEPENDENT VARIABLES				
	
ANGLE	OBJECT RELEVANCE	AGENT AGE	*M* (*sd*) RESPONSE TIME (ms)	*M* (*sd*) ACCURACY (%)	*M* (*sd*) TOTAL FIXATIONS	*M* (*sd*) PROPORTIONATE DWELL TIME	*M* (*sd*) AREA OF INTEREST TRANSITIONS

**45**	**Relevant**	**Child**	1153 (630)	98.0 (14.1)	3.83 (1.88)	65.2 (36.0)	1.52 (0.54)

		**Adult**	1180 (568)	97.6 (15.4)	3.84 (1.70)	64.8 (38.0)	1.50 (0.51)

	**Irrelevant**	**Child**	1267 (643)	98.3 (12.8)	4.10 (1.85)	61.2 (37.2)	1.58 (0.52)

		**Adult**	1170 (542)	99.1 (9.56)	3.80 (1.70)	66.3 (34.2)	1.55 (0.51)

**135**	**Relevant**	**Child**	1642 (959)	90.8 (29.0)	4.80 (2.49)	57.1 (36.1)	1.62 (0.53)

		**Adult**	1650 (917)	93.5 (24.7)	4.68 (2.27)	54.9 (38.9)	1.59 (0.54)

	**Irrelevant**	**Child**	1678 (971)	91.2 (28.3)	4.77 (2.43)	52.2 (38.4)	1.64 (0.54)

		**Adult**	1699 (942)	91.5 (27.9)	4.93 (2.49)	57.2 (35.9)	1.64 (0.51)


**Table 2:** Results from Mixed Effects Logistic Regression on Accuracy Data and Linear Mixed Effects Analysis on Response Times.


	ACCURACY			RESPONSE TIMES			
	
	ODDS RATIO	95% CI	*p*	COEFFICIENTS	95% CI	*t*-VALUE	*p*

**Fixed Effect: Angle**	0.09	0.03, 0.22	<.001	544.13	465.94, 622.33	13.64	<.001

**Fixed Effect: Avatar-age**	0.55	0.16, 1.67	.289	86.91	10.62, 163.20	2.23	0.026

**Fixed Effect: Object Relevance**	0.37	0.11, 1.04	.063	10.49	–66.01, 86.99	0.27	0.788

**Interaction: Angle × Avatar-age**	1.69	0.50, 6.23	.393	–93.70	–204.30, 16.89	–1.66	.097

**Interaction: Angle × Object Relevance**	3.64	1.13, 13.15	.030	–64.58	–175.12, 45.95	–1.15	0.252

**Interaction: Avatar-age × Object Relevance**	2.15	0.53, 9.43	0.277	–112.14	–220.34, –3.94	–2.03	0.042

**Interaction: Angle × Avatar-age × Avatar-gender**	0.33	0.06, 1.63	0.161	124.28	–32.10, 280.65	1.56	0.119

(Intercept)	219.84	87.33, 696.311	<.001	1180.64	990.81, 1370.48	12.19	<.001


**Table 3:** Results from Linear Mixed Effects Analysis on Number of Fixations, Area of Interest Transitions and Dwell Time Proportions.


	NUMBER OF FIXATIONS				AREA OF INTEREST TRANSITIONS				DWELL TIME PERCENTAGE			
		
	COEFFICIENTS	95% CI	*t*-VALUE	*p*	COEFFICIENTS	95% CI	*t*-VALUE	*p*	COEFFICIENTS	95% CI	*t*-VALUE	*p*

**Fixed Effect: Angle**	1.17	0.96, 1.37	11.21	<.001	.09	0.04, 0.14	3.53	<.001	–9.77	–14.34, –5.19	–4.19	<.001

**Fixed Effect: Avatar-age**	0.26	0.06, 0.45	2.53	.012	0.02	–0.03, 0.07	0.77	.444	–5.56	–10.05, –1.07	–2.43	.015

**Fixed Effect: Object Relevance**	0.03	–0.17, 0.22	0.26	.798	–0.05	–0.10, 0.0001	2.03	.043	–1.79	–6.29, 2.71	–0.78	0.44

**Interaction: Angle × Avatar-age**	.040	–0.69, 0.11	2.72	.007	–0.03	–0.10, 0.05	0.68	0.495	–0.06	–6.53, 6.41	–0.02	0.99

**Interaction: Angle × Object Relevance**	.027	–0.56, 0.02	1.83	.067	0.003	–0.07, 0.08	0.08	0.933	–0.41	–6.88, 6.06	–0.12	0.90

**Interaction: Avatar-age × Object Relevance**	.026	–0.54, 0.02	1.83	.068	.004	–0.07, 0.08	0.12	0.907	5.53	–0.83, 11.90	–1.71	0.09

**Interaction: Angle × Avatar-age × Avatar-gender**	0.54	0.14, 0.95	2.62	.009	.04	–0.07, 0.14	0.67	.501	1.46	–7.68, 10.61	0.31	0.75

(Intercept)	3.83	3.36, 4.31	15.82	<.001	1.55	1.44, 1.67	27.19	<.001	66.38	58.16, 74.60	15.84	<.001


### Object analysis

Before proceeding to hypothesis testing, it was important to confirm that there was no effect of object. Linear mixed effects analysis found no significant effect of object on response times, fixations, AOITs or proportionate dwell time (all *p*s > .515). Logistic regression found no effect of object on accuracy (*p* = .851).

### Response Times

**Figure 3:**
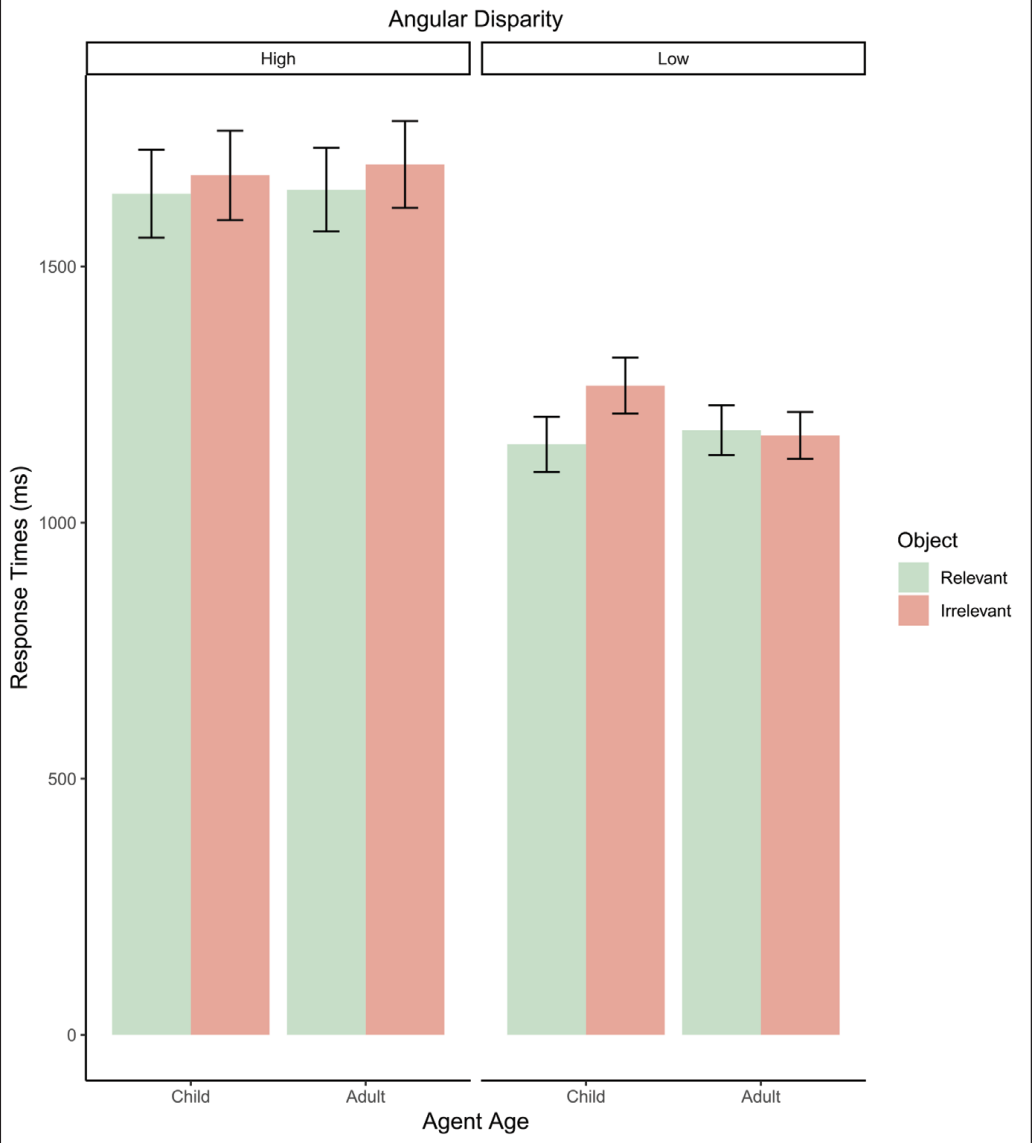
Bar plot depicting response times of all conditions. Error bars = 95% confidence intervals.

Response time analysis found a significant effect of angle (*β* = 544.13, *t*(4042) = 13.64, *p* < .001; Std. *β* = 0.66) with participants slower at higher compared to lower angles. There was also a significant effect of avatar-age (*β* = 86.91, *t*(4042) = 2.23, *p* = 0.026; Std. *β* = 0.11) with slower response times for child avatars. However, no significant effects of object relevance were found (*p* = 0.79). Analysis found a significant interaction between avatar-age and avatar-object relevance (*β* = 112.14, *t*(4042) = 2.03, *p* = 0.042; Std. *β* = 0.14); Figure 3 and related post-hoc comparisons report no significant effect of object relevance for adult avatars (*p* = .44) but do reveal a significant effect of object relevance for child avatars (*p* = .013) with slower response times for irrelevant compared to relevant objects (see Figure 3). No other interactions were significant (all ps > .097).

### Accuracy

**Figure 4:**
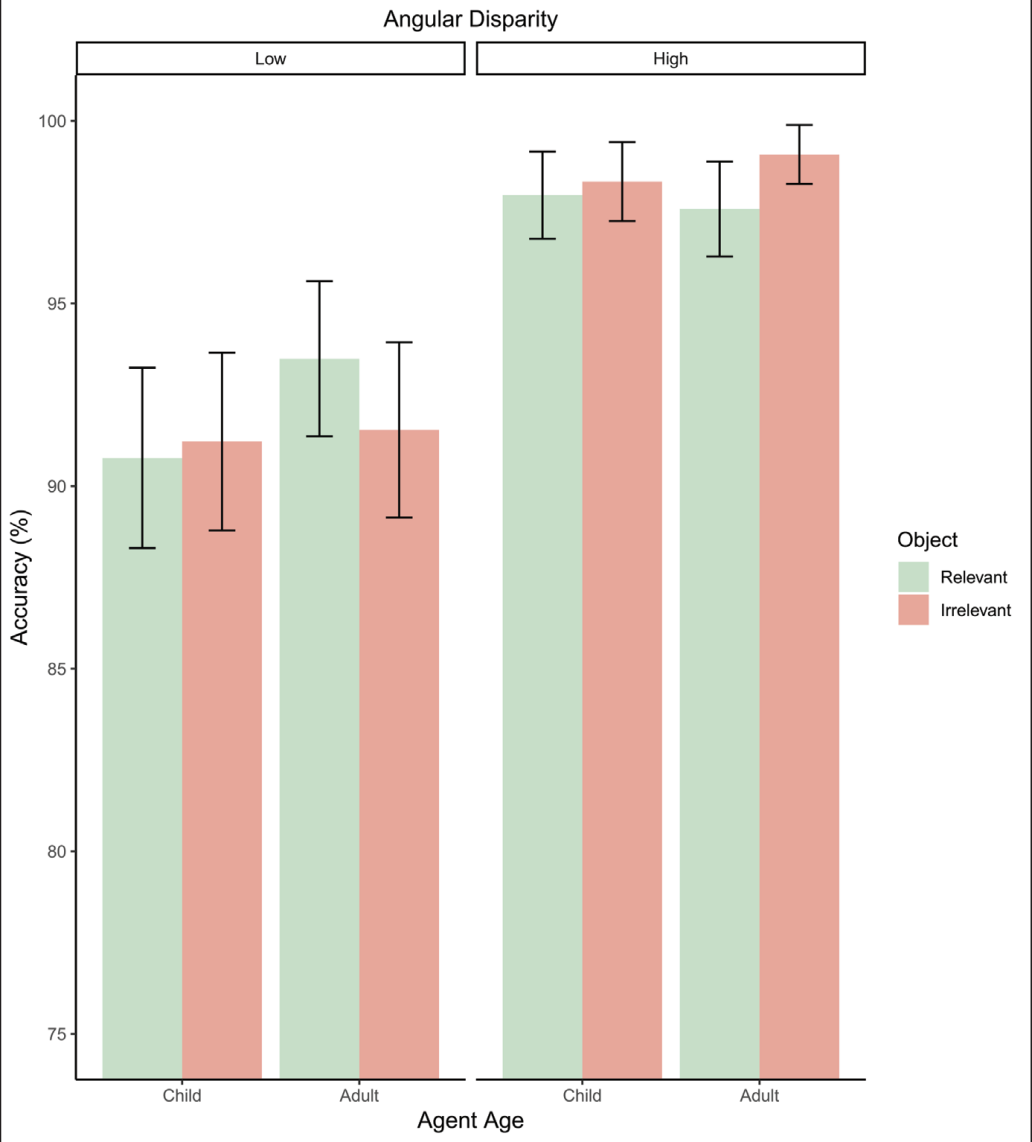
Bar plot depicting accuracy as a percentage for all conditions. Error bars = 95% confidence intervals.

Accuracy analysis found a significant fixed effect of angle (*OR* = .09, *p* < .001) with participants less accurate at 135° than at 45°. No effects of age (*p* = 0.29) or object relevance were found (*p* = .063). Analysis revealed significant interaction between angle and relevance (*OR* = 3.64, *p* = 0.03) with higher accuracy for relevant objects at low angular disparity but higher accuracy for irrelevant objects at high angular disparity. Though, post-hoc comparisons reveal no significant differences between object relevance conditions at low (*p* = .09) or high angles (*p* = .49) (see Figure 4). No other interactions were significant (all ps > .16).

### Total Fixations

**Figure 5:**
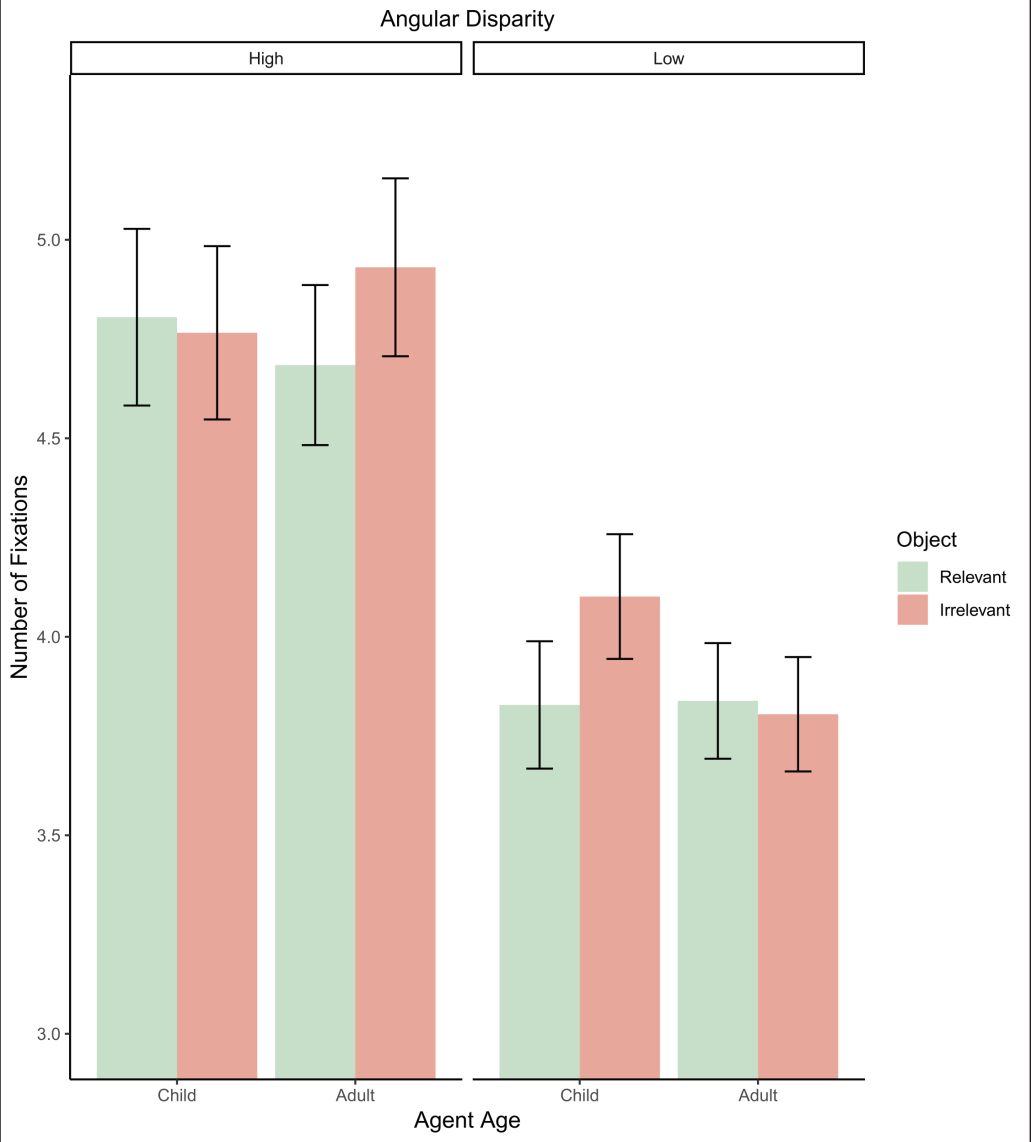
Bar plot depicting the three-way interaction between avatar-age and object-relevance on number of fixations. Error bars = 95% confidence intervals.

Number of fixation analysis found a significant main effect of angle (*β* = 1.17, *t*(4043) = 11.21, *p* < .001; Std. *β* = 0.54) with more fixations at higher angles compared to lower angles. Furthermore, a significant effect of avatar-age was found (*β* = 0.26, *t*(4043) = 2.53, *p* = 0.012; Std. *β* = 0.12) with more fixations made when representing the perspective of a child. However, there was no significant effects of object relevance (*p* = 0.80). Analysis found an interaction between angular disparity and avatar-age (*β* = 0.40, *t*(4043) = 2.72, *p* = 0.007; Std. *β* = 0.19) with more fixations for child avatars at low angles but no difference at high angles. No other two-way interactions were significant (both *p*s > .067). However, analysis revealed a significant three-way interaction effect (*β* = 0.54, *t*(4043) = 2.62, *p* = 0.009; Std. *β* = 0.25). Figure 5 and post-hoc comparisons reveal that at low angles object relevance affects child avatars (*p* = .020) with increased fixations when the object is irrelevant but has no impact on adult avatars (*p* = .798). Conversely, at higher angles object relevance does not impact child avatars (*p* = .710) but does affect adult avatars (*p* = .023) with more fixations when an irrelevant object is presented.

### Proportionate Dwell Time

**Figure 6:**
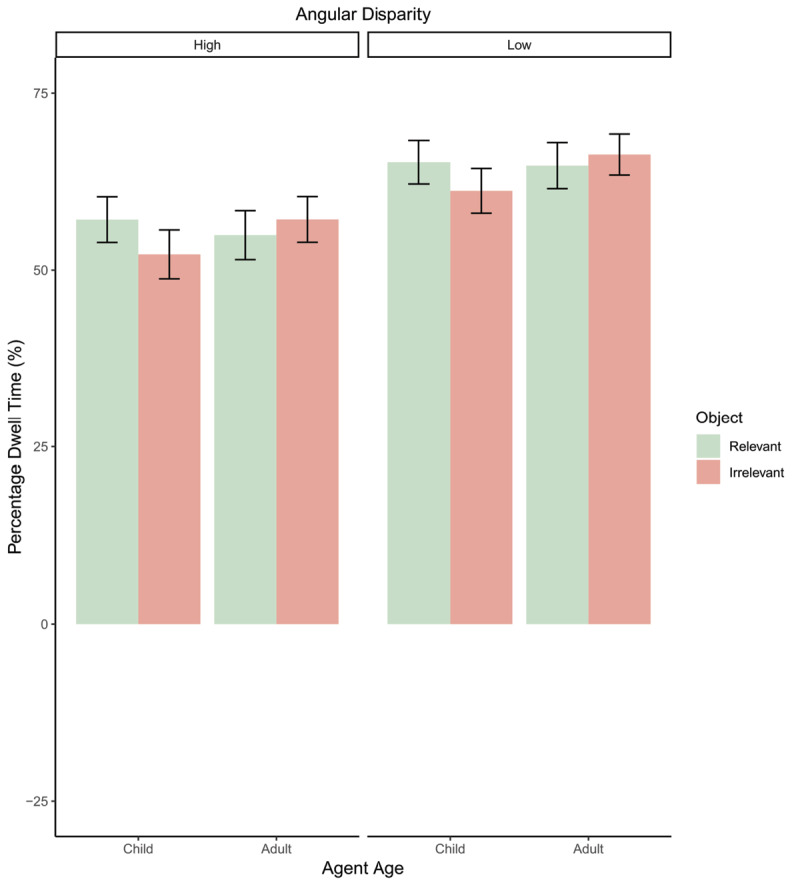
Bar plot depicting dwell time percentages for all conditions. Error bars = 95% confidence intervals.

Dwell time percentage analysis (depicted in Figure 6) found a significant effect of angle, with more time spent looking at the avatar during high angular disparity judgements (*β* = –9.77, *t*(4042) = –4.19, *p* < .001; Std. *β* = –0.26). There was also a significant effect of avatar-age, with more time spent looking at the object with adult-avatars than child-avatars (*β* = –5.56, *t*(4042) = –2.43, *p* = 0.015; Std. *β* = –0.15). No significant effect of object relevance (*p* = .44) or any significant interaction effects were observed (all *p*s > .09).

### Area of Interest Transitions

**Figure 7:**
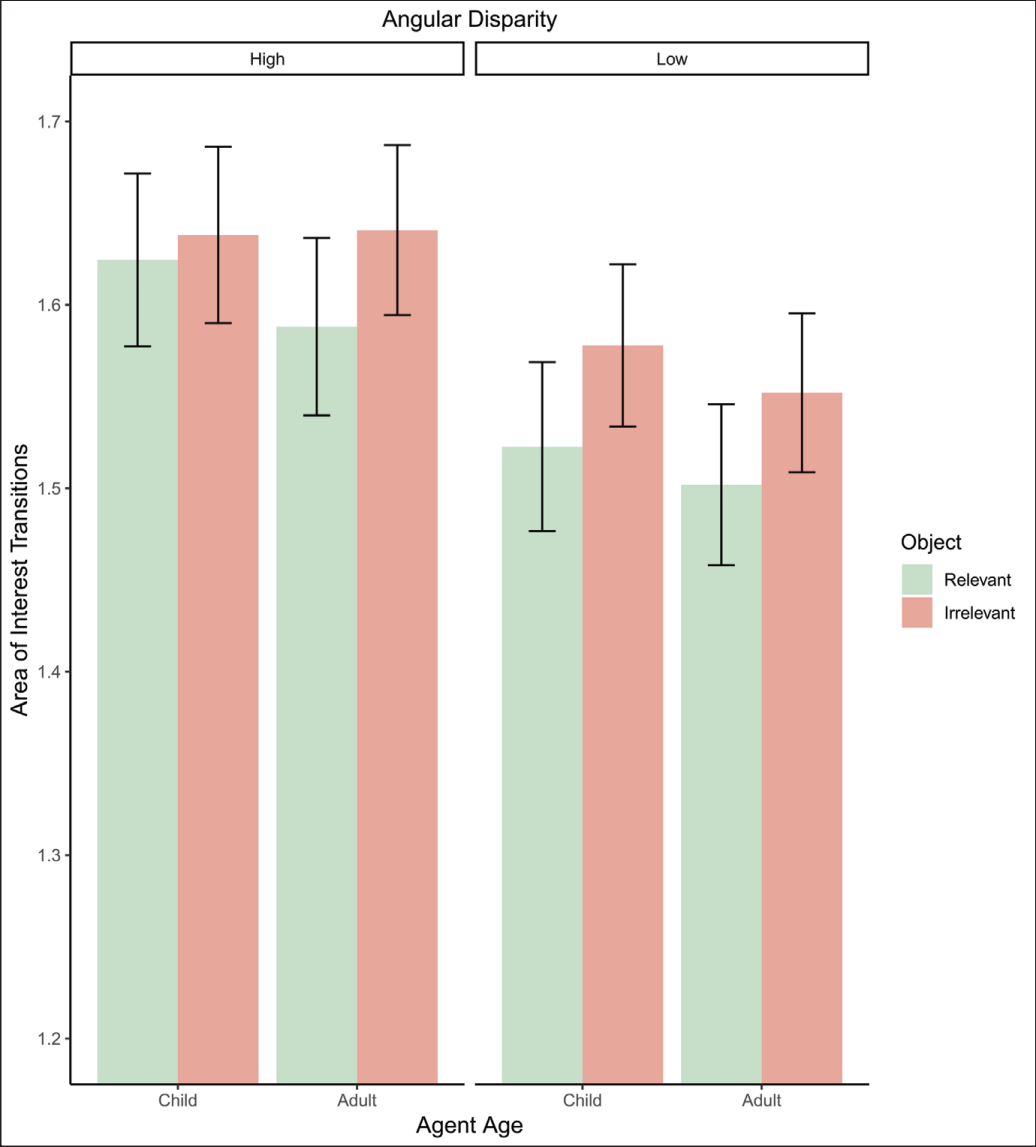
Bar plot depicting area of interest transitions for all conditions. Error bars = 95% confidence intervals.

Area of interest transition analysis (depicted in Figure 7) found a significant effect of angle (*β* = 0.09, *t*(4043) = 3.53, *p* < .001; Std. *β* = 0.18) with more AOITs at higher angles than at lower angles (see Figure 2). A significant effect of object relevance was observed (*β* = –0.05, *t*(4043) = 2.03, *p* = 0.043; Std. *β* = 0.10) with more AOITs for irrelevant than relevant objects (see Figure 3). There was no observed effect of avatar-age (*p* = .44). No interactions were significant (all *p*s > .50).

## Discussion

The current study had three main aims. First, to supplement behavioural and neurophysiological distinctions between low and high angular judgements with attention data. Second, to further examine the effects of avatar characteristic in L2-VPT using eye-tracking to measure the number of fixations, the number of transitions between agents and target objects, and the proportion of time spent fixating on the agents and objects. Third, to explore whether stronger thematic relationships between agent and object impact L2-VPT similarly to how they affect gaze judgements (Gong et al., 2021). Replicating previous work, results suggest that compared to 45°, perspective taking at 135° slowed response times and increased error-rates. The current results further our understanding by demonstrating that accompanying the longer response times and reduced accuracy, there is also a greater number of fixations, an increased number of transitions between agents and objects, and a greater consideration of the agent’s position in space. Furthermore, extending the current understanding of agent characteristic effects in L2-VPT, results showed that age affected the proportion of time spent dwelling on the avatar compared to the object (with more time spent on the object when the avatar was an adult). Moreover, age also interacted with object relevance to modulate response times. Also, age interacted with object relevance and angular disparity with object irrelevance increasing fixations for child avatars at low angles but increasing fixations for adults at high angles. Finally, incongruent agent-object relationships resulted in a greater number of transitions between areas of interest.

A corpus of work supports the distinction between processing level-2 visual perspectives at low and high angular disparity (Janczyk, 2013) and our findings of slower response times and lower accuracy at higher angles support a body of existing research relating increasing angular disparity to greater cognitive effort (Michelon & Zacks, 2006; Kessler & Thomson, 2010; Surtees et al., 2013a). Previous neurophysiological findings report additional temporoparietal and executive network activity at higher angular disparity, responsible for inhibiting the egocentric view, selecting the alternate position, and manipulating the visuospatial information (Seymour et al., 2018). Seymour et al. contrast the bottom-up, externally driven processing at low angles when self and other perspectives are aligned to the need for rerouting visual information at higher angles to form an internal representation of the world. Our proportionate dwell time findings reveal that increased attention is paid to the avatar’s position that occurs when participants inhibit the egocentric view and assume the alternate position. Furthermore, the increased fixations and area of interest transitions that we observed appear to reflect the greater processing of visual information (e.g., Blythe et al., 2009; Young & Hulleman, 2013; Molitor et al., 2014) that is required to form an internal representation at high angular disparities, supporting similar findings in L1-VPT (Wu & Keysar, 2007; Ferguson et al., 2017). In contrast, at low angles, accurate judgments can be achieved using egocentric visuospatial representations (Briscoe, 2009; Foley et al., 2015) which is reflected in reduced proportionate dwell time to the avatar and less agent-object transitions that are unnecessary for efficient perspective taking at low angles.

Evidence is increasing that agent attributes such as humanness, group-status and age affect the computation of visual perspectives (Todd et al., 2011; Ferguson et al., 2018; Ye et al., 2020). Accordingly, the current research demonstrated a significant effect of age on proportionate dwell time, with similarly aged avatars attended to less than dissimilarly aged avatars. This could reflect similarity facilitation reported in L2-VPT (Ye et al., 2020) where an easier ‘step’ into the ingroup other’s position is accompanied by less attentional engagement. Initially, this appears contrary to the ingroup interference observed in L1-VPT (Savitsky et al., 2011; Simpson & Todd, 2017; Ferguson et al., 2018) and spatial navigation (Todd et al., 2011) where dwell times could have been expected to increase because of egocentric interference. However, the stimuli in Ye et al. (2020) are most similar to the task used in the current study, which highlights important differences between tasks. Egocentric interference impacts L1-VPT and an interpersonal communication task used by Todd et al. (2011) but the current task and Ye et al. (2020) employ non-communicative L2-VPT tasks and similarity appears to facilitate the effort required to respond correctly. Thus, the specific differences between L1- and L2-VPT tasks which result in different avatar-characteristic effects are worth further examination.

Whilst acknowledging that salient characteristics modulate attention in previous research (e.g., Wu et al., 2012; Kawakami et al., 2014; Denkinger & Kinn, 2018; Strickland-Hughes et al., 2020), we advise caution interpreting our main effects of age as evidence of outgroup classification effects. First, the main effects of age are arguably better explained by the significant interactions. For instance, although a significant main effect of age was found for number of fixations inside AOIs, the three-way interaction demonstrates the age relationship was precise and nuanced. Specifically, at low angles irrelevant objects increased the number of fixations for child avatars, whereas at high angles irrelevant objects led to more fixations for adult avatars. Similarly, for the main effect of age on response times, the two-way interaction with object-relevance showed that child avatars looking at relevant objects were processed more quickly than those looking at irrelevant objects, with object relevance having no impact for adult agents. Second, and more fundamentally, participant attention was focused significantly more on the object regardless of avatar-age. Therefore, whether avatar-age was, or even could have been, processed independently of the object and their relationship remains unclear. Thus, considering the small effects of age in previous work (Ford et al. 2023), we hesitate to suggest that age had a significant *independent* impact in the current experiment. Further clarification is required using a task isolating avatar-age and where attention is not explicitly directed towards the object, perhaps by varying stimuli configuration (e.g., non-centrally appearing agents and objects).

As hypothesised, agent-object incongruence increased the number of AOI transitions, extending the known influence of thematic relationships on language comprehension (Estes et al., 2011) to include attentional allocation in sociocognitive processes. One explanation could be that more efficient attention allocation results simply from the efficient processing of semantically related information in a general representation mechanism (Xu et al., 2018). Here, incongruent pairings increase the demand on attentional mechanisms because their saliency is highlighted which may disrupt processing automaticity (Chaigneau et al., 2009; Estes et al., 2011; Kalénine et al., 2012). A second explanation of agent-object effects could be intentionality processing. Despite ‘full-blown’ ToM not being required in terms of explicit reasoning or action description (Butterfill & Apperly, 2013), the combination of thematic relationships and gaze-cues may represent minimal requirements for attributions of intentionality (Castiello, 2003; Becchio et al., 2008; Chaigneau et al., 2009), or at-least increase the likelihood of a mentalising-based route to perspective judgments. Thus, considering the close relationship between ToM and VPT (Dumontheil et al., 2010; Hamilton et al., 2009), the representation of an intentional mind may be one specific mechanism that affects attention allocation in L2-VPT. If so, the current findings represent the first step towards understanding how implicit inferences of intentionality arising from the interaction between individual attributes and social-contextual cues affect attentional processes involved in perspective representation. This also has consequences for predicting behaviour and how unlikely relationships between agents and objects are processed by inferential and interpretative processing networks (Brass et al., 2007; Richardson & Saxe, 2020).

Object relevance was also found to interact with avatar-age. Whilst object relevance did not affect response times for adult avatars, for child avatars, age-relevant objects reduced response times and age-irrelevant objects increased response times. This suggests that while less interference is experienced from incongruent cues when paired with an in-group member, additional social cues facilitate or disrupt perspective representation of outgroup others. Importantly, real-world experience is important for developing thematic relationships (Estes et al., 2011) as well as our bias to perceive outgroups as more homogenous (Boldry et al., 2007; Konovalova & Le Mens, 2020). As such, the current findings suggest that experience may influence our conceptual flexibility. Specifically, the experience we have with in-group others increases conceptual flexibility of their interactions with objects in the environment. Conversely, the lesser experience we have with out-groups constrains our conceptions of what objects they are likely to interact with, and the more likely agent-object pairings are to influence concurrent processing. Given the lack of previous research examining outgroup schemas and agent-object interaction, our findings offer initial support that congruent pairings, which confirm rigid ideas about outgroup-object relationships, may reduce processing demand relative to incongruent pairings. Conversely, ingroup heterogeneity may extend to object interaction and agent-object incongruencies do not significantly impact processing difficulty. Thus, although most VPT research isolates specific task cues, the current findings highlight the importance of integrating multiple cues, especially if we are to develop predictive theories about VPT in everyday social interactions.

The interaction between avatar-age and object relevance visible in eye-tracking data was modulated by angular disparity. At low angles, where perspective taking can rely on an egocentric frame of reference (Wang et al., 2016), object incongruence increased fixations for child agent trials. Here, dissimilar others who share our perspective present a conflict of group categorisation and coupled with agent-object incongruency, which may violate outgroup homogeneity assumptions, these inconsistencies increase attentional engagement (Henderson et al., 1999; Võ & Henderson, 2009). Conversely, similarly aged others offer little interference from categorisation processes or from agent-object incongruency perhaps due to in-group heterogeneity biases. Conversely, at high angular disparity, object incongruence increased fixations for adult agent trials. At high angles, perspective taking is effortful and involves a deliberate mental self-rotation (Kessler & Thomson, 2010) with increased focus on the agent because their location indicates when self-rotation is complete (Ward et al., 2020). In another L2-VPT task, Ye et al. (2020) concluded that dissimilar avatars produce less interference as a function of outgroup dehumanisation. Similarly, we propose that with the increased focus on mental self-rotation and spatial processing, social agency is more easily disregarded for outgroup members and the agent-object incongruency less salient. Yet, with ingroup agents, participants may suffer greater egocentric interference because of the combination of ingroup status, mental self-rotation, and perspective conflict. This is in line with other findings from L1-and-L2-VPT where ingroup status interferes with processing (Savitsky et al., 2011; Simpson & Todd, 2017; Todd et al., 2011; Ferguson et al., 2018) as well as reports where conflicting perspectives increase fixation counts in L1-VPT (Wu & Keysar, 2007).

### Limitations

On all trials, objects and agents were located in predictable positions. Real-world social cues are often dynamic with regular gaze shifts, varying distances and changing agent-object relationships. To better resemble real-world social processing, future eye-tracking research could vary room layouts or present stimuli as to encourage naturalistic viewing behaviour (Kim et al. 2020). Manipulating age also has potential limitations. First, participant’s experience with children may modulate responses to agent-object pairings (Estes et al., 2011) with the undergraduate sample recruited potentially having less experience with young children than the general population. However, agent age was not manipulated for specific age-related hypotheses. Instead, age was a pragmatic choice to represent a general outgroup of everyday relevance, discernible from salient characteristics (Ferguson et al., 2018). Relatedly, we are unable to ascertain how participants categorised agents or conceptualised agent-object relationships. For instance, Ferguson et al. (2018) suggest that adults assume a reduced mental capacity for children which reduces perspective saliency. Thus, perhaps reduced readiness for mentalising is a crucial factor and future research is required to understand precisely what moderates differential attentional processing towards groups and agent-object relationships.

### Future directions

Our angular disparity findings raise an interesting question regarding attention allocation required for different types of L2-VPT. Previous work distinguishes between visual and spatial forms of L2-VPT and finds that judging whether a ‘6’ or ‘9’ appears on the left or right is more effortful than whether the agent ‘sees’ a ‘6’ or ‘9’ (Surtees et al., 2013a; Surtees et al., 2013b). Based on our findings, increased effort could be a function of increased information gathering when it is necessary to assess object-agent relationships on *spatial* perspective taking. Conversely, for *visual* perspective tasks where targets are easily represented from any view, participants need only consider the approximate agent position. Future eye-tracking research could elucidate attentional processes underlying this visual-spatial distinction. Furthermore, our hypothesis that implicit mentalising about intentionality affects L2-VPT should be examined. For example, replicating the current method but grouping participants by autistic traits would be pertinent, as has been conducted in gaze-cueing research (Morgan et al. 2021). Also, understanding how intentionality judgments impact perspective-taking across the lifeitalic are warranted considering the development and decline of perspective taking and theory-of-mind in younger and older age groups (Klindt et al., 2017; De Lillo & Ferguson, 2023).

## Conclusion

Evidence that agent-object relationships significantly impact cognitive effort and attention allocation in L2-VPT demonstrate the role of perspective representation in understanding intentionality and predicting behaviour. Our results are the first to demonstrate the effects angular disparity on attention allocation in L2-VPT, extending the current cognitive and neurophysiological evidence (Kessler & Thomson, 2010; Seymour et al., 2018). Our findings are also the first evidence of social cues affecting attention orienting in L2-VPT and build on previous work in L1-VPT (Wu & Keysar, 2007; Ferguson et al., 2017; Wei et al., 2020) and gaze-cueing (Kawakami et al., 2018). Specifically, we extend the findings reported for isolated agent-characteristics (Savitsky et al., 2011; Todd et al., 2011; Simpson & Todd, 2017; Ferguson et al., 2018; Ye et al., 2020) to agent-object relationships. More generally, despite the fundamental spatial processing at the heart of L2-VPT tasks (Kessler & Thomson, 2010; Ward et al., 2020), our findings suggest that human attributes of agents are being considered along with other socio-contextual cues. Understanding what specific contexts and which specific cues affect visuospatial processing will support the development of integrative accounts of perspective taking. These accounts can incorporate underlying processes, person-attributes, task demands and relevant sociocontextual information, allowing researchers to make theory-led predictions about when representing other’s experiences is facilitated or disrupted.

## Data Accessibility Statement

Raw data available at: https://doi.org/10.17605/OSF.IO/8GDM6.

## Footnotes

For clarity, including stimuli here controls for the potential variability introduced by individual stimuli.
